# Field Testing of the Use of Intake24—An Online 24-Hour Dietary Recall System

**DOI:** 10.3390/nu10111690

**Published:** 2018-11-06

**Authors:** Maisie K. Rowland, Ashley J. Adamson, Ivan Poliakov, Jennifer Bradley, Emma Simpson, Patrick Olivier, Emma Foster

**Affiliations:** 1 Human Nutrition Research Centre, Institute of Health & Society, Newcastle University, Newcastle upon Tyne NE2 4HH, UK; Ashley.adamson@ncl.ac.uk (A.J.A.); Jen.bradley@ncl.ac.uk (J.B.); Emma.simpson@ncl.ac.uk (E.S.); Emma.foster@ncl.ac.uk (E.F.); 2 Open Lab, School of Computing Science, Newcastle University, Newcastle upon Tyne NE4 5TG, UK; Ivan.poliakov@ncl.ac.uk (I.P.); Patrick.olivier@ncl.ac.uk (P.O.)

**Keywords:** Intake24, online dietary assessment, 24-h food recall

## Abstract

Dietary assessment is important for monitoring and evaluating population intakes. Online tools can reduce the level of participant burden and the time taken to complete records, compared with other methods. The study aimed to field test an online dietary recall tool (Intake24) to test the suitability for collecting dietary information in Scottish national surveys and to develop the system based on feedback and emerging issues. Previous Scottish Health Survey participants, aged 11+ years, were invited to complete Intake24 and provide feedback about it. Of those who agreed to take part, 60% completed at least one recall. Intake24 was found to be user-friendly, enjoyable to use, and easy to follow and understand. Users agreed they would like to use Intake24 often, (44% compared with 15% who disagreed) and >75% felt the system accurately captured their dietary intakes. The main challenge reported was finding foods within the database. Of those completing fewer recalls than requested, the majority reported that they believed they had completed the required number or reported not receiving emails requesting they complete a further recall. Intake24 was found to be a user-friendly tool allowing dietary assessment without interviewer presence. Feedback indicated the method for recall reminders needs to be refined and tailored.

## 1. Introduction

Dietary assessment is important for monitoring and evaluating dietary intakes of a population. The use of online dietary recalls as a method of dietary assessment has been found to reduce the level of burden on participants and reduce the time taken to complete records, compared with other assessment methods [1]. The multiple pass method (MPM) [2] allows detailed dietary information to be obtained whilst avoiding the use of time consuming and burdensome methods such as the weighing of foods [1]. Online tools allow large sample sizes to be targeted and there is no need for the presence of an interviewer; they ensure that coding is consistent and enable much faster and cheaper dietary analysis to be conducted, with only minimal manual data entry required [3]. There is evidence that 24-h recalls and food diaries provide more accurate and less biased estimates of usual dietary intake than food frequency questionnaires (FFQs) and that two to four recalls are better than FFQs for estimating usual dietary intake of all but those food groups which are rarely consumed [4]. There are a number of online dietary tools worldwide including myfood24 (UK), ASA24 (USA) and YANA-C (Belgium), all of which are based on an automated MPM [5,6,7].

Intake24 is an online 24-h dietary recall tool that allows users to self-report their dietary intake. The system is based on the automated MPM and has over 2400 food photographs of more than 100 foods for portion size estimation based on the portion sizes of foods reported in the UK National Diet and Nutrition Surveys (NDNS) [8]. These photos have been validated in a feeding study and a relative validation against four-day weighed intake records [9,10]. The database contains more than 2300 foods and is regularly expanded to include new foods. It has a ‘missing foods’ tool allowing users to highlight food/drinks that are not in the database and there is a bespoke spelling correction system which handles most cases of misspelled food names. There is a function allowing participants to add their own recipes and a ‘Make your own sandwich/salad’ function enabling participants to add fillings and other items to their sandwich/salad. The system was developed by an iterative process involving four cycles of user-testing and development [11] and it was compared against interviewer-led recalls in a relative validation study [12].

Intake24 has a video tutorial explaining the features of the system and there are contextual help buttons describing the functions of the different aspects.

The aim of the study was to test the usability of Intake24 in the general population aged 11–65+ years (as it was originally developed for 11–24 year-olds) and to further develop the system based on user feedback and emerging issues. It also aimed to assess the suitability of Intake24 as a tool for collecting dietary information in Scottish national surveys.

## 2. Materials and Methods

Ethical approval for the study was granted by the Newcastle University Faculty of Medical Sciences Ethics Committee (00875/2015).

In collaboration with ScotCen Social Research (a part of NatCen Social Research), 1000 participants who had previously taken part in the Scottish Health Survey (SHeS), 50% in 2013 and 50% in 2014 aged 11 years and over, were invited to take part in the study. The population sample was stratified by age, gender and deprivation using the Scottish Index of Multiple Deprivation (SIMD) [13]. A minimum of 20 people in each separate stratum were selected with over-sampling in sub-sets of the population in which digital technology adoption and frequency of use is known to be low, particularly older people and those living in the most deprived areas [14]. One thousand participants were approached in order to ensure sufficient numbers to allow meaningful analysis by the stratified sample.

Introductory letters were sent in the post and these were followed up by a phone call from the NatCen Telephone Unit to establish whether or not the individual wanted to take part and if so, whether they had internet access. Those who agreed to take part were asked to complete Intake24 on four occasions (non-consecutively) over a 10-day period, including both week (two days) and weekend days (two days). Participants were notified of their days and prompted to complete their recall by either text messages, emails or letters depending on the details they provided. For participants aged 11–15 years, all contact was with their parent/carer. Participants who completed Intake24 on all four days were sent a £20 Post Office voucher. Date of birth, gender, SIMD and body mass index (BMI) were obtained from the SHeS dataset for those consenting to participate in the study.

Feedback on the usability of the system along with suggestions for improvements and details of any issues encountered were collected using SurveyMonkey^®^ (see Tables S1–S4). It also enabled feedback on the newer functions of the system, such as the sandwich and recipe functions, to be collected. The questionnaire was linked to Intake24 and participants were asked to complete the questionnaire after their final dietary recall.

Participants that initially agreed to take part, but stopped completing the recalls, were sent a short questionnaire via email link or were telephoned to ascertain their reasons for stopping. Those who agreed to take part but did not log on were telephoned and asked what had prevented them from completing the recalls.

Statistical significance testing was performed using SPSS Version 21 to establish whether or not there were any differences between groups in either response to the telephone unit stage or the recall stage. The approach chosen was two-tailed logistic regression which enabled the testing of between group differences, for example if there was any statistical difference in numbers agreeing to take part in the study by the stratified groups (gender, age group, etc.), or any differences in the number of people completing recalls by these stratified groups. *p* values less than 0.05 were deemed significant and are marked with an asterisk (*). Results of recall completion are presented with 95% confidence intervals. Sentiment analyses was performed on the free text comment section in the feedback questionnaire and responses were categorised into either negative, mixed, neutral or positive comments. Google analytics was used to gain information about technology usage.

## 3. Results

### 3.1. Participant Response

**Figure 1:**
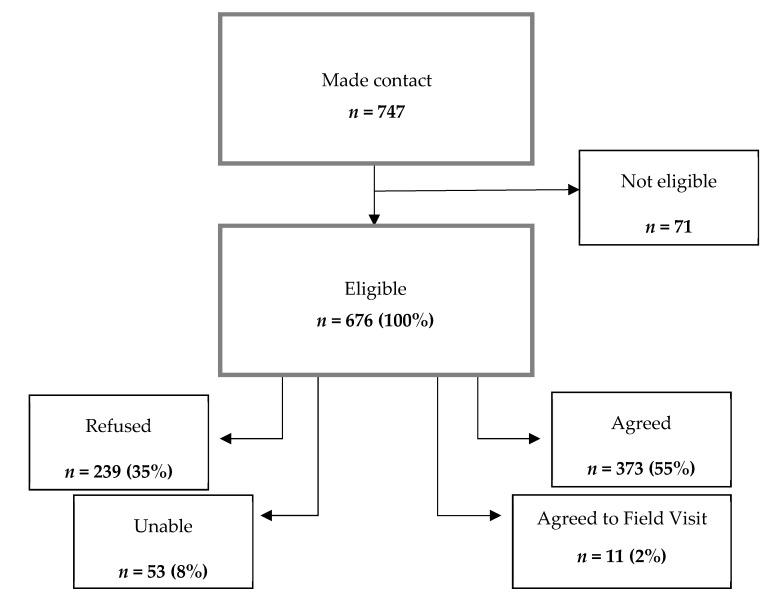
Participant contact and response.

The NatCen Telephone Unit was able to make contact with 747 participants, 74.7% of the participant sample (*n* = 1000). Of these, 71 (9.5%) were considered ineligible and were excluded from the response rates. Those classed as ineligible were those whose physical or mental health circumstances had changed since completing the Scottish Health Survey (SHeS) and those who were not able to take part as they were not available during the fieldwork period. Of those individuals who were eligible (*n* = 676), 55.1% agreed to take part, 1.6% agreed to a field visit whereby a researcher visited them to help complete their first recall), 35.4% refused to take part, and 7.8% were classed as unable to take part (Figure 1).

Table 1 below represents the reasons for participants being “unable to take part”. One individual could be coded to more than one reason. The most common reasons for being unable to take part in the study were linked to the lack of internet connectivity; “not having internet access” or “not having any devices linked to the Internet” (69% of answers). The reason “age” was described for those participants who felt they were too old to take part. Other reasons included “not being interested”, “not having time to take part”, and “not taking part for health reasons”.

Analyses were performed to assess the statistical significance of difference in response rate by demographic group (gender, age group, BMI, or SIMD) for the “agreed”, “refused” and “unable” groups. There were no significant differences between the outcomes by gender or by SIMD. There was a statistically significant difference by age group whereby the youngest participants (aged 11–16 years) were more likely to take part than the other age groups, particularly those aged 65+ years with 81% and 27% agreeing respectively (*p* value < 0.001). Similarly, the 65+ year age group was the most likely to refuse to take part with 54% refusing (see Supplementary Material for further information).

### 3.2. Demographics of Those Who Completed a Recall

Participant demographics of those eligible and those agreeing to take part (which includes those who agreed to a field visit) are shown in Table 2. There were equal numbers of male and female participants who were classed as eligible to take part, however slightly more males than females agreed to take part (203 males cf. 181 females). Of the participants agreeing to take part, 47% were in the healthy weight category (*n* = 165) with only 2% in the underweight category (*n* = 6). There was an almost equal split between the quintiles of SIMD (Table 2).

### 3.3. Number of Recalls Completed

Of the participants who agreed to take part, 230 (~60% of those who agreed) completed at least one recall and within these participants, 132 completed four or more recalls (~34% of those who agreed) (Table 3).

Recall completion rates of those who agreed to take part in the study by gender, age group, BMI and SIMD classification are shown in Table 4, Table 5, Table 6 and Table 7. There were no differences in completion rates between gender (95% confidence intervals (CIs) male: 34.3–47.8, female: 32.4–46.6) (*p* value: 0.741), age group (CI 11–16 years: 35.4–52.8, 17–24 years: 22.8–43.0, 25–64 years: 30.0–47.4, 65+ years: 34.5–59.3) (*p* value 0.256) or BMI (CI Under-weight 16.7–83.3, Healthy weight: 28.8–43.4, Over-weight: 34.7–53.1, Obese: 33.5–58.5, Morbidly obese: 15.6–62.5) (*p* value 0.564) (Table 4, Table 5 and Table 7 below). However, there was a difference in completion rates by SIMD quintiles (CI SIMD1: 40.7–61.6 and SIMD5: 17.4–36.9, *p* value 0.019) (Table 6). Those that were in the most deprived SIMD quintile were significantly less likely to complete a recall (Table 6).

### 3.4. Technology Use

Google Analytics was used to identify platforms (mobile vs. desktop), operating systems and browsers. The most popular browsers used were Chrome (~60%) and Safari (~25%), with recent versions of Internet Explorer (version 10 and 11) also being frequently used (~9%); indicating that a significant portion of the user base are well supported. Sixty-six percent of users logged in using either laptops or desktops, 22% used a smart phone, and 11% used tablets.

### 3.5. All Participant Feedback on Intake24

In total 245 participants logged on to Intake24 (this includes 15 participants who did not go on to complete a recall) of these, 182 (74%) completed the feedback questionnaire using SurveyMonkey^®^. In addition to the fixed choice questions, there was a section allowing optional free text for participants to add further comments (see Supplementary Material). A sentiment analysis was performed on the free text answers allowing the classification of statements into ‘negative’, ‘neutral’, ‘positive’, and ‘mixed’ categories (Table 8). Examples of some of the positive quotes included “Easy to use, enjoyable and user friendly” (female, 25–64 years) and “I honestly enjoyed taking part…Visual representation of what I had worked well for me and helped me give more accurate information” (female, 11–16 years). Examples of negative quotes included “Visuals were dull, e.g., plain white plates on a white background” (female, 11–16 years) and “If possible, minimize questions being asked” (female, 11–16 years). An example of a mixed quote included ‘I thought it was very visual and easy to pick up but it took me longer I would of liked’ (male, 11–16 years).

**Figure 2:**
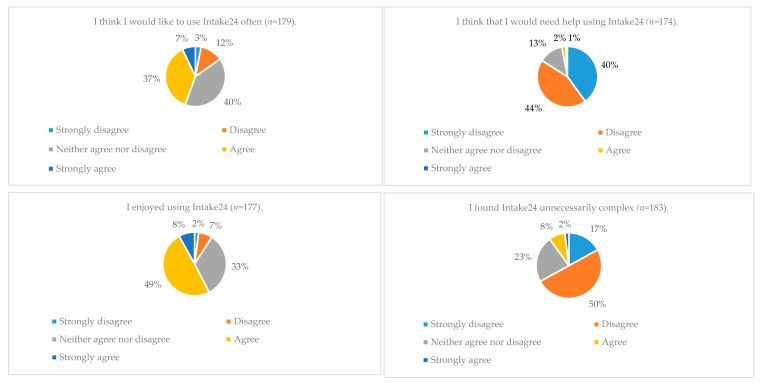
Participant feedback from fixed choice answers in questionnaire.

From the fixed choice answer questions, it was found that almost half (44%) of answers given agreed that they would like to use Intake24 often, compared with 15% that disagreed. Over 67% of answers disagreed that the system was unnecessarily complex or had too many inconsistencies and over 80% agreed that the system was easy to follow and understand. Users also found Intake24 enjoyable to use, with 57% of answers agreeing with the statement ‘I enjoyed using Intake24′ and just 9% disagreeing with this statement (Figure 2).

The majority of answers (84%) disagreed that help would be required using Intake24 with only 3% of answers agreeing that help would be needed. More than 75% of answers agreed that Intake24 accurately captured dietary intakes and 79% of answers agreed that Intake24 could be completed in a reasonable time.

### 3.6. Feedback from Those Completing Fewer than four Recalls

Feedback from those completing fewer than four recalls was obtained from 34 (37%) participants out of the 93 who were contacted by email and phone call (there were no email or phone details for five participants). Participants were asked to choose the reason(s) they did not fully complete their allocated recalls. Participants were able to give more than one answer. Of the responses, 28% indicated ‘I thought I had fully completed the survey’, 18% indicated that email reminders were missed, another 18% opened their emails too late for the allocated day, and 9% were away or had no Internet access so were unable to complete their recalls. The remaining 17% indicated that participants either struggled accessing the system or decided not to complete further recalls because they had lost interest or found it took too long (Table 9).

### 3.7. Timings of Recalls

The average time to complete recalls based on those who completed four recalls was 14 min, whereas analysis of those completing two days showed the completion time was slightly longer at 16 min on average.

### 3.8. Help Requests

There were 17 help requests from participants throughout the study. The most common issue was that participants had trouble logging in due to typing their username and password incorrectly (*n* = 10). Five participants had trouble finding a food and completing a recall and two were confused about which day to complete their recall. The majority (88%) of these issues were resolved in under 10 min.

## 4. Discussion

### 4.1. Response and Reminders

Of those classed as eligible to take part in this study, 55.1% agreed to take part, with 59.9% of those who agreed, completing at least one recall, and 34.4% completing four recalls. This gave a lower completion rate than anticipated (we had hoped for around a 50% response rate based on the NDNS rolling program response in Scotland [15]) which could be partly attributed to the recruitment and chasing strategy used.

Participants were asked only once to complete their recalls on each allocated day; no reminders were sent to encourage participation if they didn’t submit a recall. In addition, reminders were not sent directly to participants under 16 years, but instead to their parent/guardian. This could have reduced the likelihood of the participants getting the notification. However, although lower than anticipated, the recruitment rate compares favourably to similar research, where SHeS participants who had agreed to be a part of further research were invited to take part in a dietary assessment validation study using food frequency questionnaires and non-weighed food diaries; only 9% of participants agreed to take part, with just 6% completing the study [16]. Feedback from non-completers found a substantial number of participants either forgot about the study or did not see the emails. Additional reminders may reduce these issues whilst remaining inexpensive. Increasing participation through the use of reminders has been demonstrated by Christensen et al., in a Danish health survey where it was found that multiple reminders led to an increase in participant response [17]. The use of telephone calls or text messages as reminders, in particular, may increase participation whilst reducing the risk of emails being easily overlooked if, for example they are misdirected into a participant’s ‘junk folder’. A Finnish study by Tolonen et al., reported that text message reminders increased participation compared to those that didn’t receive a text reminder and research by Breen et al., concluded that telephone reminders increased response rates [18,19]. It is hypothesised that by increasing the number of reminders sent via text message, email and phone call the completion rate would increase. In particular, personalised reminders such as follow-up notifications could be sent if a participant doesn’t log in on their allocated day which might increase participation. It is also noted that the field test was conducted over just a two month period over the summer, where approximately 15% of participants indicated that although they agreed to take part, they were actually unable to take part due to being away; this would be less of an issue in a study of longer duration. Participation rates in studies have been shown to be lower in people from lower socio-economic groups [18], and as anticipated, the response and completion rates differed by level of deprivation, with lower response rates in the sub-sets of the population in which digital technology adoption and frequency of use is known to be low [14]. It is thought that additional support via telephone calls for those groups, in particular, may increase response and completion rates.

### 4.2. Usability and Developments

In terms of the usability of Intake24, there were just seventeen participant requests for help from the study team throughout the field testing. The majority of these were easily resolved, however the recurring issues with log in details suggest that for large scale surveys, using a unique link, rather than ask the user to enter a username and password may help to alleviate such issues. From the Google analytics data, the majority (66%) of participants used a computer to complete Intake24, however with around 33% of participants using either a smart phone or tablet, future developments could be made to create an App specifically designed for these devices. The developments identified through the field testing have been used to improve the usability and accuracy of the system. These included the addition of prompts, so when commas, ‘/’ or ‘&’ are entered into the system, the user is asked whether these are ‘separate foods’. Previously, when many items were listed on one line, this sometimes resulted in only one of those foods or drinks being entered into the system. Through use of the ‘missing foods’ function, new foods were identified and added into the database (this is a continuous process and therefore ensures the system is as comprehensive as possible). In addition to identifying new foods, the missing food function can be used to highlight foods that are in the database, but that users were unable to locate. Meta-data for these foods (synonyms and brand names) can then be added in order to make them easier for participants to find and therefore minimise the number of foods that require manual coding. These changes are likely to improve the accuracy of the nutrient data and to reduce the time taken for manual coding. Furthermore, a ‘Frequently Asked Questions’ page has been added along with a more concise video tutorial including instructions on how to use the ‘Recipe Tool’. In addition, due to the majority (75%) of participants stating they would like to receive dietary feedback from their recalls, an optional feature of providing personalised dietary feedback on completion of a recall has been developed.

The feedback questionnaire used in this study allowed for extensive usability feedback to be obtained from participants. However, unfortunately due to the rewording of some of the questions in the survey, we were unable to use participant answers to rate Intake24 using the Standard Usability Scale [20]. This is a limitation of the study, meaning it cannot be compared to other tools rated using this scale. In future we would aim to include these questions in feedback questionnaires regarding Intake24 enabling such comparisons to be made [20].

## 5. Conclusions

The overall feedback on Intake24 was very positive and allowed identification of key aspects of the system which required adaption. Although the completion rate was lower than expected, possible reasons for this have been identified and it is likely that completion rates could be increased using additional reminders. Key aspects of development were identified based on extensive user feedback, enabling the system to be further improved.

## Figures and Tables

**Table 1:** Participant reasons for being ‘unable’ to take part.

Reasons	Answers Obtained (*n* = 99)	
	*n* =	%
No internet access	41	41
No devices linked to internet	28	28
Not comfortable/competent using computers/tablets/smart phones	24	24
Going away or in hospital during fieldwork	3	3
Age	3	3

**Table 2:** Participant demographics of those eligible (*n* = 676) and those who agreed (*n* = 384) by gender, age, BMI and SIMD.

Demographic		Eligible (*n* = 676) ^1^		Agreed (*n* = 384) ^1^	
		*n* =	%	*n* =	%
Gender	Male	343	51	203	53
	Female	333	49	181	47
Age Group	11–16 years	151	22	123	32
	17–24 years	121	18	81	21
	25–64 years	183	27	120	31
	65+ years	221	33	60	16
Body mass index (BMI) classification	Under weight	7	1	6	2
	Healthy weight	255	38	165	47
	Over weight	195	29	110	31
	Obese	126	19	59	17
	Morbidly obese	24	4	14	4
Scottish Index of Multiple Deprivation (SIMD) Quintiles	1 (Most deprived)	174	26	86	22
	2	111	16	64	17
	3	136	20	81	21
	4	126	19	76	20
	5 (Least deprived)	129	19	77	20

^1^ For the BMI data, there are 69 cases missing in the “eligible” group, and 30 cases missing in the “agreed” group.

**Table 3:** Response rates for those who were eligible and who agreed.

Number of Recalls	Eligible (*n* = 676)		Agreed (*n* = 384)	
	*n* =	%	*n* =	%
0	446	66.0	154	40.1
4+	132	19.5	132	34.4
Any	230	34.0	230	59.9

**Table 4:** Recall completion by gender of those agreed.

Number of Recalls	Male (*n* = 203)			Female (*n* = 181)			
	*n* =	%	CI	*n* =	%	CI	*p* Value
0	83	40.9	34.3–47.8	71	39.2	32.4–46.6	0.741
4+	70	34.5	28.2–41.3	62	34.3	27.7–41.5	0.963
Any	120	59.1	52.2–65.7	110	60.8	53.4–67.6	0.741

**Table 5:** Recall completion by age of those who agreed. CI: confidence interval.

Number of Recalls	Age Group (Years)												
	11–16 (*n* = 123)			17–24 (*n* = 81)			25–64 (*n* = 120)			65+ (*n* = 60)			*p* Value
	*n* =	%	CI	*n* =	%	CI	*n* =	%	CI	*n* =	%	CI	
0	54	43.9	35.4–52.8	26	32.1	22.8–43.0	46	38.3	30.0–47.4	28	46.7	34.5–59.3	0.256
4+	40	32.5	24.8–41.3	32	39.5	29.5–50.5	41	34.2	26.2–43.1	19	31.7	21.2–44.5	0.726
Any	69	56.1	47.2–64.6	55	67.9	57.0–77.2	74	61.7	52.6–70.0	32	53.3	40.7–65.5	0.256

**Table 6:** Recall completion by SIMD quintile of those who agreed.

Number of Recalls	SIMD Quintile															
	1 (Most Deprived) (*n* = 86)			2 (*n* = 64)			3 (*n* = 81)			4 (*n* = 76)			5 (Least Deprived) (*n* = 77)			*p* Value
	*n* =	%	CI	*n* =	%	CI	*n* =	%	CI	*n* =	%	CI	*n* =	%	CI	
0	44	51.2	40.7–61.6	30	46.9	35.0–59.1	32	39.5	29.5–50.5	28	36.8	26.8–48.2	20	26.0	17.4–36.9	0.019 *
4+	22	25.6	17.4–35.9	22	34.4	23.8–46.8	30	37.0	27.2–48.1	26	34.2	27.2–48.1	32	41.6	31.1–52.9	0.305
Any	42	48.8	38.4–59.3	34	53.1	40.9–65.0	49	60.5	49.5–70.5	48	63.2	51.8–73.2	57	74.0	63.1–82.6	0.19 *

* *p* values less than 0.05 were deemed significant.

**Table 7:** Recall completion by BMI classification of those who agreed (Note: there are missing data for 30 participants).

Number of Recalls	BMI Classification															
	Under-Weight (*n* = 6)			Healthy Weight (*n* = 165)			Over-Weight (*n* = 110)			Obese (*n* = 59)			Morbidly Obese (*n* = 14)			*p* Value
	*n* =	%	CI	*n* =	%	CI	*n* =	%	CI	*n* =	%	CI	*n* =	%	CI	
0	3	50.0	16.7–83.3	59	35.8	28.8–43.4	48	43.6	34.7–53.1	27	45.8	33.5–58.5	5	35.7	15.6–62.5	0.564
4+	3	50.0	16.7–83.3	65	39.4	32.2–47.1	35	31.8	23.8–41.1	16	27.1	17.3–39.8	4	28.6	11.1–56.2	0.374
Any	3	50.0	16.7–83.3	106	64.2	56.6–71.2	62	56.4	46.9–65.3	32	54.2	41.5–66.5	9	64.3	37.5–84.4	0.564

**Table 8:** Sentiment analysis from free text question in feedback questionnaire.

Sentiment	*n* = 72	%
Negative	11	15
Neutral	24	33
Positive	29	40
Mixed	8	11

**Table 9:** Participant reasons for not fully completing recalls (34 participants, 57 responses).

Reason Given	Number of Responses (*n* = 57)	% ^1^
Thought I had fully completed the survey	16	28
Don’t remember seeing any more emails	10	18
Opened the email too late to complete for allocated day	10	18
Did not receive any more emails	7	12
Away/Holiday/No (or problems with) Internet access ^1^	5	9
Didn’t enjoy doing it	3	5
Unable to access the website	2	4
It took too long	1	2
Lost interest	1	2
Could access the website but was unable to log on	1	2
Had enough after completing 1, 2 or 3 recalls	1	2
Changed mind about taking part	0	0
Didn’t like that it was online	0	0

^1^ Discrepancies in percentages due to rounding.

## Supplementary Materials

The following are available online at http://www.mdpi.com/2072-6643/10/11/1690/s1, Table S1: Difference in percentage for response outcomes of those classed as eligible (*n* = 676) by Gender, Table S2: Difference in percentage for response outcomes of those classed as eligible (*n* = 676) by Age group, Table S3: Difference in percentage for response outcomes of those classed as eligible (*n* = 676) by BMI classification, Table S4: Difference in percentage for response outcomes of those classed as eligible (*n* = 676) by SIMD quintile.
