# Supplementary material for: Field Testing of the Use of Intake24—An Online 24-Hour Dietary Recall System
Source: Nutrients. 2018 Nov 6;10(11):1690. doi: 10.3390/nu10111690 (PMC6266941; doi:10.3390/nu10111690)
Supplement: Supplementary file 1 [file nutrients-10-01690-s001.zip › Supplementary Material/Additional response outcome tables by demographics- Supplementary Material.docx]

**Supplementary Material**

| Outcomes | Gender | | |
| --- | --- | --- | --- |
|  | Male | Female | **P value** |
| Agreed | 59% | 55% | 0.206 |
| Refusal | 34% | 37% | 0.314 |
| Unable | 7% | 8% | 0.589 |
| *n*= | 343 | 333 |  |
| *%^1^* | 100% | 100% |  |

**Table S1.** Difference in percentage for response outcomes of those classed as eligible (n=676) by Gender.

^1^ Discrepencies in percentages due to rounding

**Table S2**. Difference in percentage for response outcomes of those classed as eligible (n=676) by Age group

| Outcomes | *Age (years)* | | | |  |
| --- | --- | --- | --- | --- | --- |
|  | 11-16 | 17-24 | 25-64 | 65+ | P value |
| Agreed | 81% | 67% | 65% | 27% | <0.001* |
| Refusal | 18% | 31% | 30% | 54% | <0.001* |
| Unable | 1% | 2% | 5% | 19% | <0.001* |
| *n=* | 151 | 121 | 183 | 221 |  |
| *%^1^* | 100% | 100% | 100% | 100% |  |

^1^ Discrepencies in percentages due to rounding

**Table S3**. Difference in percentage for response outcomes of those classed as eligible (n=676) by BMI classification

| Outcomes | *BMI classification* | | | | | P value |
| --- | --- | --- | --- | --- | --- | --- |
|  | UW | HW | OW | OB | MO |  |
| Agreed | 86% | 65% | 56% | 47% | 58% | 0.012* |
| Refusal | 14% | 30% | 34% | 43% | 33% | 0.118 |
| Unable | 0% | 5% | 9% | 10% | 8% | <0.001* |
| *n=* | 7 | 255 | 195 | 126 | 24 |  |
| *%^1^* | 100% | 100% | 100% | 100% | 100% |  |

^1^ Discrepencies in percentages due to rounding

**Table S4**. Difference in percentage for response outcomes of those classed as eligible (n=676) by SIMD quintile

| Outcomes | *SIMD Quintile* | | | | | P value |
| --- | --- | --- | --- | --- | --- | --- |
|  | 1 | 2 | 3 | 4 | 5 |  |
| Agreed | 50% | 58% | 59% | 60% | 60% | 0.257 |
| Refusal | 38% | 35% | 37% | 32% | 34% | 0.840 |
| Unable | 13% | 7% | 4% | 8% | 6% | 0.070 |
| *n=* | 174 | 111 | 136 | 126 | 129 |  |
| *%^1^* | 100% | 100% | 100% | 100% | 100% |  |

^1^ Discrepencies in percentages due to rounding
