# Supplementary material for: Field Testing of the Use of Intake24—An Online 24-Hour Dietary Recall System
Source: Nutrients. 2018 Nov 6;10(11):1690. doi: 10.3390/nu10111690 (PMC6266941; doi:10.3390/nu10111690)
Supplement: Supplementary file 1 [file nutrients-10-01690-s001.zip › Supplementary Material/Feedback questionnaire - Supplementary Material.pdf]

Feedback Questionnaire Intake24

Please answer the following questions related to your experience of using INTAKE24.

Thank you for providing feedback, we will feed this into further development of INTAKE24.

1. For each of the following statements, mark one box that best describes your reactions to INTAKE24.

	Strongly disagree	Disagree	Neither agree nor disagree	Agree	Strongly agree
I think I would like to use INTAKE24 often.	○	○	○	○	○
I found INTAKE24 unnecessarily complex.	○	○	○	○	○
I think that I would need help using INTAKE24.	○	○	○	○	○
I thought there was too much inconsistency in INTAKE24.	○	○	○	○	○
I **don't** think people would learn to use INTAKE24 very quickly.	○	○	○	○	○
I **didn't** feel very confident using INTAKE24.	○	○	○	○	○

2. For each of the following statements mark one box that best describes your reactions to INTAKE24.

	Strongly disagree	Disagree	Neither agree nor disagree	Agree	Strongly agree
INTAKE24 accurately captured my dietary information.	◯	◯	◯	◯	◯
INTAKE24 was easy to follow and understand.	◯	◯	◯	◯	◯
INTAKE24 is visually appealing.	◯	◯	◯	◯	◯
I was able to complete INTAKE24 in a reasonable time.	◯	◯	◯	◯	◯
I enjoyed using INTAKE24.	◯	◯	◯	◯	◯

Any issues when finding foods in INTAKE24?

3. Did you have any problems when finding foods in INTAKE24?

- ○ Yes
- ○ No

Example of finding a food from the returned list.

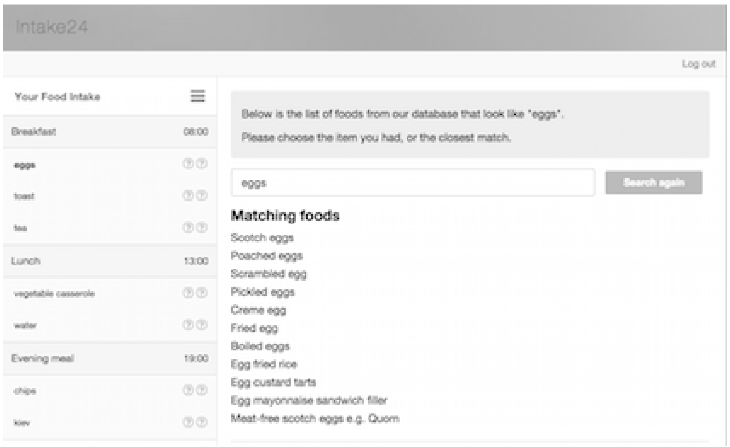

4. If yes, what problems did you have when trying to find a food in INTAKE24?

5. Any further comments or suggestions for improvement?

Did you have issues with portion size slelection in INTAKE24?

6. Did you have any problems with the portion size selection in INTAKE24?

- ◯ Yes
- ◯ No

Example of the portion size selection.

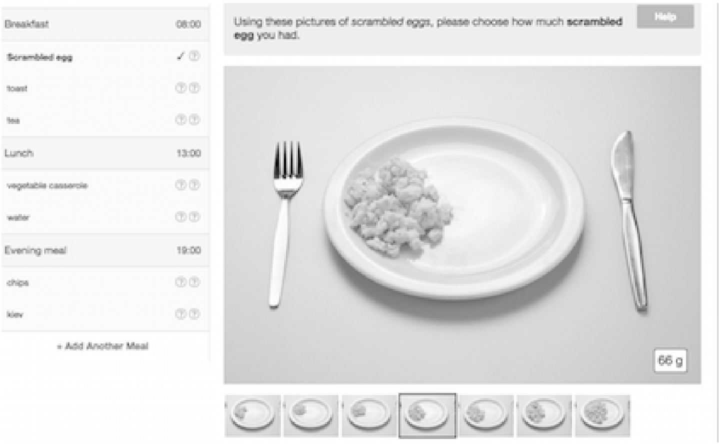

7. If yes, what problems did you have with the portion size selection?

Did you have any problems when adding recipes in INTAKE24?

8. If you added any homemade recipes, did you have any problems using this part of INTAKE24?

- ◯ Yes
- ◯ No
- ◯ I did not enter any recipes

9. If yes, what problems did you have when adding recipes?

Did you have any problems when adding missing foods in INTAKE24?

10. Did you have any problems when entering missing food items in INTAKE24?

- ◯ Yes
- ◯ No
- ◯ I found all the food items I was looking for in INTAKE24.

Example of a missing food entry.

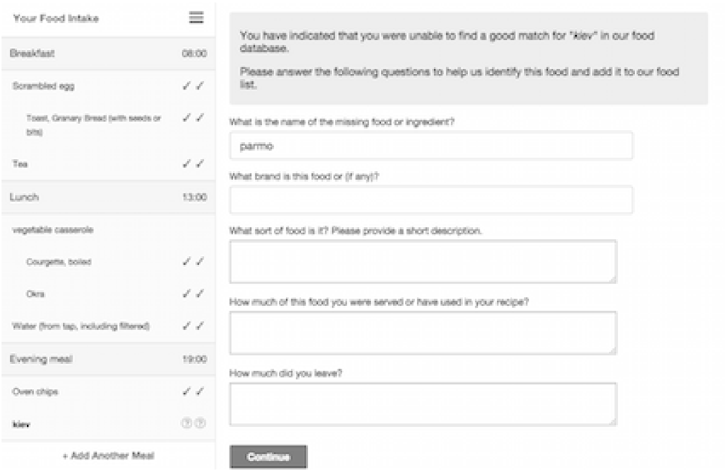

11. If yes, what problems did you have when entering missing food items in INTAKE24?

12. Do you have any further comments regarding INTAKE24, including your overall experience of using the system or any improvements you would like to see in a future system?

Future food surveys

13. In future surveys, would you be interested in receiving feedback from INTAKE24 on your diet?

- ○ Yes
- ○ No

14. In future food surveys, what would your preferred mode of contact be for notifying you that you are due to complete INTAKE24 on a particular day?

- ○ Text
- ○ Email
- ○ Phone call
- ○ Letter (postal)

Many thanks for taking part in this study – we really appreciate you providing us with feedback about INTAKE24. If you have completed all 4 recalls we will send you your Post Office voucher (that you exchange for cash at your local Post Office) by 31st August. If you have still not received your voucher by 14th September then please call us on 0800 526 397.
